# High‐Speed, Maneuverable, and Terrain‐Adaptive Micro‐Robot with Tree Frog‐Inspired Bionic Feet

**DOI:** 10.1002/advs.202514807

**Published:** 2025-11-26

**Authors:** Weizhi Zhao, Shijia Li, Kaiwen Zhang, Wanyu Zhang, Danyang Liu, Weidong Fang, Yanxin Zhai, Kaibo Lei, Liwen Zhang, Huawei Chen, Tiantong Xu

**Affiliations:** ^1^ Research Institute of Aero Engine Beihang University Beijing 100191 China; ^2^ Research Institute of National Excellence for Engineers Beihang University Beijing 100191 China; ^3^ School of Energy and Power Engineering Beihang University Beijing 100191 China; ^4^ School of Mechanical Engineering & Automation Beihang University Beijing 100191 China; ^5^ Taihang Laboratory Chengdu 610299 China

**Keywords:** aero‐engine detection, bionic feet, controllability, high‐speed, micro‐robot

## Abstract

Micro‐robots hold promise for complex tasks such as fault diagnosis and emergency response, where conventional detection methods are limited by size and maneuverability constraints. However, their adaptability in complex environments remains insufficient owing to reduced propulsion efficiency. This study proposes a 7.5 mm micro‐robot actuated by an electromagnetic linear motor and integrated with tree frog‐inspired bionic feet (MRBF) to enhance traction and locomotion performance. The bionic feet are fabricated via angled photolithography to realize the microstructured design. The integration of bionic feet enables the MRBF to reach a maximum velocity of 39 body lengths per second (BL s^−1^) on dry surfaces and 28.5 BL s^−1^ on wet surfaces, representing improvements of 75% and 40%, respectively, compared to the non‐bionic version. The MRBF can also climb inclines of up to 21.8°, nearly doubling its original climbing limit of 11°, demonstrating a considerably enhanced slope‐climbing capability. A dual‐MRBF system is specifically designed to achieve rapid and controllable turning, attaining angular velocities of ≈311° s^−1^. When equipped with a micro‐camera, MRBFs are successfully deployed for in situ blade inspection within the confined intake duct of a micro‐aero‐engine. This strategy provides a scalable framework for adaptive, high‐performance systems in aerospace inspection, soft robotics, and autonomous sensing.

## Graphical abstract

A 7.5 mm micro‐robot integrates tree frog‐inspired bionic feet with an electromagnetic linear motor to achieve high‐speed locomotion, slope climbing, and precise turning. It reaches 39 body lengths per second and ascends 30° slopes. The system demonstrates strong potential for inspection tasks in confined and complex environments, such as micro‐aero‐engines.

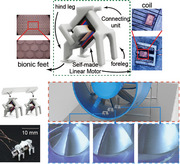

## 1 Introduction

Detection operations are essential in various scenarios, including the inspection of tiny cracks and damages in aero‐engines,^[^ ^1^, ^2^, ^3^, ^4^ ^]^ navigation through the complex, curved pipelines used in oil and gas transportation,^[^ ^5^, ^6^ ^]^ and access during rescue missions in narrow terrains caused by earthquake damage.^[^ ^7^, ^8^ ^]^ These detection tasks face significant challenges in confined environments with adverse conditions such as complex terrain, moisture, oil contamination, and dust.^[^ ^9^, ^10^, ^11^ ^]^ Such environments impose stringent requirements on the motion stability and terrain adaptability of detection machines, such as endoscopic. Although modern industrial endoscopes are equipped with controllable bending sections and high‐resolution optics, their inspection capability is inherently constrained by the number and position of borescope ports.^[^ ^12^, ^13^ ^]^ These ports are distributed only at a few fixed axial and circumferential locations owing to structural and aerodynamic design considerations, which restrict both insertion pathways and viewing angles.^[^ ^14^ ^]^ Consequently, endoscopic inspection typically provides partial or spot‐check coverage of the interior, leaving large regions, such as compressor blade inner surfaces, stator vanes, and inter‐blade passages, inaccessible.^[^ ^15^, ^16^, ^17^ ^]^ To address these issues, studies have been mostly focused on the development of micro‐robots, which are characterized by miniaturized designs and multiple degrees of freedom. Micro‐robots are designed to autonomously crawl through confined, curved, and branching flow paths to directly access deep and geometrically complex regions that are beyond the reach of flexible endoscopes.^[^ ^18^ ^–^ ^20^ ^]^

However, the adaptability of existing micro‐robots to complex environments remains insufficiently demonstrated. First, most micro‐robots have only exhibited locomotion performance on dry surfaces. For example, a shape memory alloy (SMA)‐driven micro‐robot (10 mm in length), designed to mimic cheetah‐like locomotion, achieved an impressive speed of 42.8 body lengths per second (BL s^−1^) and a turning rate of 482° s^−1^.^[^ ^21^ ^]^ However, its capabilities on wet or irregular surfaces were not evaluated. Similarly, an SMA‐driven micro‐robot (13 mm in length and 30 mg in weight) underwent motion testing at various actuation frequencies, but its locomotion performance under wet conditions was not verified.^[^ ^22^ ^]^ An all‐optical driven soft crawler can reportedly crawl at speeds of up to 2.25 BL min^−1^, perform turning and obstacle avoidance, and manipulate or transport objects approximately twice its own weight. However, its locomotion performance under complex conditions, such as wet or low‐friction surfaces, has not yet been evaluated.^[^ ^23^ ^]^

Second, most millimeter‐scale micro‐robots exhibit only linear locomotion capabilities. For instance, an electromagnetically actuated crawling micro‐robot (5 mm in length) achieved a maximum speed of 20.2 BL s^−1^ but was restricted to linear movement.^[^ ^24^ ^]^ A 3.4 mm‐long electrostatically driven micro‐robot that mimicked flea locomotion reached an extreme speed of 46 BL s^−1^, yet its movement remained imprecise and primarily linear.^[^ ^25^ ^]^ A piezoelectric alloy‐driven micro‐robot (30 mm in length) demonstrated the advantages of high‐speed (20.2 BL s^−1^), strong pressure resistance, and good climbing capability,^[^ ^26^ ^]^ but it was limited to single‐degree‐of‐freedom motion. The PiezoClimber can rapidly climb vertical (90°) substrates at a speed of 1.4 BL s^−1^, exhibiting excellent wall‐climbing capability, which is attributed to its bioinspired adhesive surfaces. However, its inability to perform controlled turning maneuvers limits its motion flexibility.^[^ ^27^ ^]^ Another bioinspired footed soft robot can traverse terrains with varying roughness, slope, and surface wetness, and can operate over a wide temperature range, while carrying loads up to 50 times its own body mass. Despite these impressive load‐bearing and environmental adaptability characteristics, its overly simplified and lightweight structural design results in insufficient steering capability and reduced motion controllability.^[^ ^28^ ^]^

Moreover, some micro‐robots could only adapt to flat terrains and lacked slope‐climbing capabilities. For example, although soft electromagnetic micro‐robots achieved up to 70 BL s^−1^ on specific surfaces, their climbing performance on inclined slopes was not verified.^[^ ^29^ ^]^ An SMA‐driven micro‐robot (57 mm in body length) was capable of climbing slopes only up to 15°.^[^ ^30^ ^]^ Similarly, a wireless‐enabled, electromagnetically driven micro‐robot (20 mm in length) could only climb slopes of 6°, which is insufficient for many practical applications.^[^ ^31^ ^]^ In summary, most existing millimeter‐scale micro‐robots support only single‐mode movement and lack multi‐degree‐of‐freedom mobility. Furthermore, very few designs validate motion performance in complex environments such as wet or irregular surfaces. Therefore, there is an urgent need for highly integrated micro‐robots that combine compact size, high‐speed movement, terrain adaptability, and controllable turning capabilities.

For micro‐robots, improving speed, terrain adaptability, and turning ability is critical. However, such enhancements often result in increased size and structural complexity. Conventionally, the most direct approach to improving locomotion performance is by enhancing actuation power. Meanwhile, increasing foot friction can also significantly improve movement speed and enable better adaptability in complex environments.^[^ ^32^ ^]^ At present, few studies in the literature address the integration of foot structure design in micro‐robots. Robots without specialized foot structures often show performance degradation, especially when operating on complex surfaces or climbing slopes.^[^ ^33^ ^]^ In nature, tree frogs exhibit exceptional terrain adaptability.^[^ ^34^ ^]^ Inspired by such biological models, incorporating bionic feet into micro‐robot designs can further enhance locomotion performance. At the same time, achieving complex motion modes presents a major challenge. With special mechanical designs, it is possible to implement sophisticated functions using simplified mechanisms, allowing micro‐robots to maintain complex mobility while adhering to miniaturized structural constraints.^[^ ^35^, ^36^, ^37^ ^]^

Despite notable progress in micro‐robot development, a comprehensive comparison reveals that most existing systems involve trade‐offs between speed, adaptability, and maneuverability. For example, SMA‐driven micro‐robots often feature high power density and compact structures but suffer from low energy efficiency and slow thermal response, making them less suitable for sustained or energy‐constrained engineering applications. Electrostatic and piezoelectric actuators offer fast response times and lightweight designs, but typically lack sufficient force output and terrain adaptability. Electromagnetic actuators provide better controllability and power output but may require more complex integration and higher power input. Moreover, a common limitation across many designs is the absence of multi‐degree‐of‐freedom motion and poor adaptability to wet, inclined, or irregular surfaces, conditions frequently encountered in real‐world environments.

Herein, we present a micro‐robot with bionic feet (MRBF) that achieves rapid locomotion, robust terrain adaptability, and controllable turning. Specifically, to balance miniaturization with high power output, an electromagnetic linear motor at the microscale is fabricated using a self‐developed and easily implementable processing method. Bionic feet, inspired by the adhesive structures of tree frogs, are integrated into the foot design to enhance both terrain adaptability and locomotion efficiency. The influence of various environmental conditions on the robot's performance is systematically investigated. Additionally, a modular design enables controllable motion through the assembly of a dual‐robot configuration. The system's practical potential is further explored through real‐world applications, such as in situ inspection within the confined spaces of aero‐engines.

## 2 Results and Discussion

### 2.1 Structure Design and Locomotion Analysis

**Figure 1:**
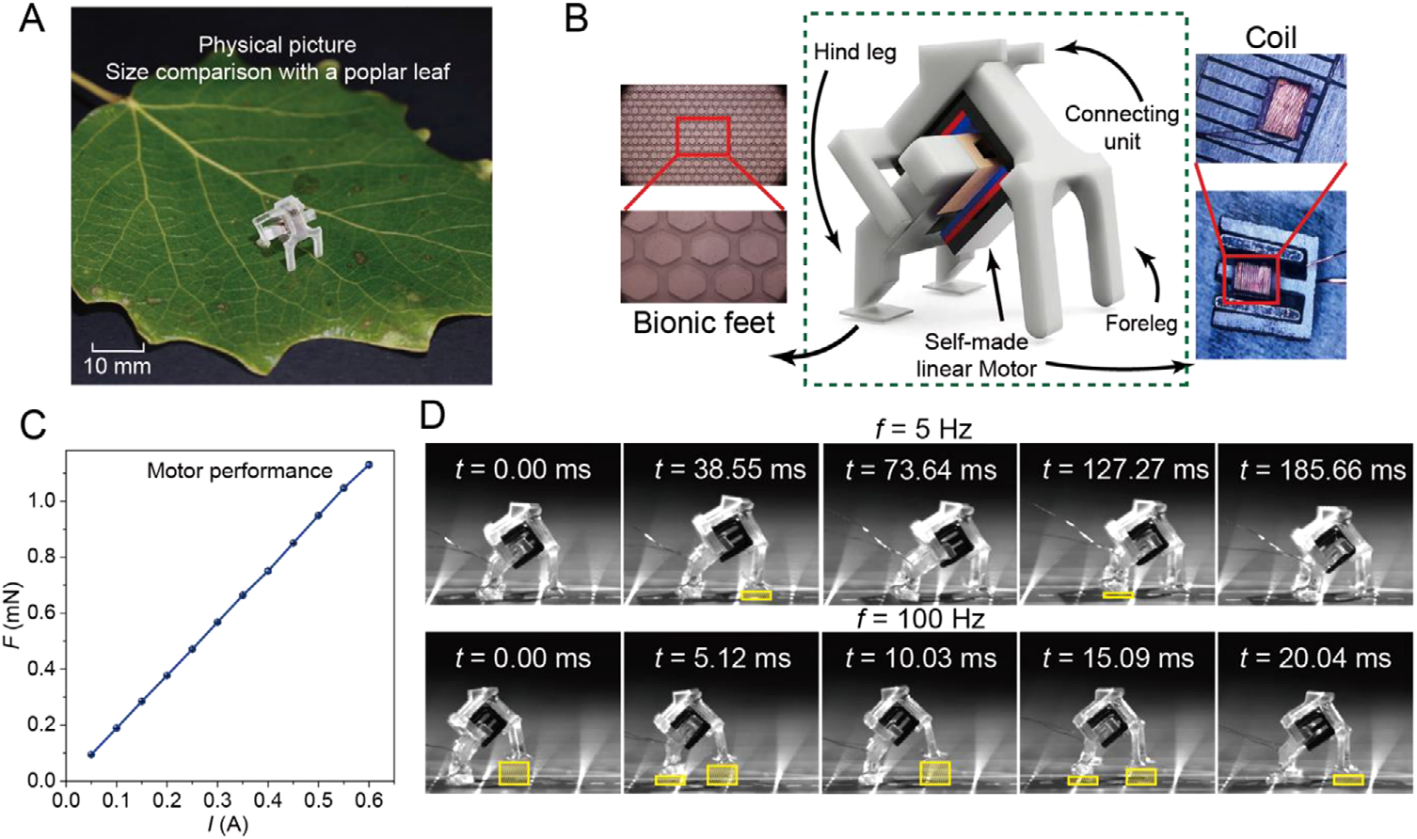
Robot structure composition and locomotion analysis. A) Optical photograph of the robot resting on a poplar leaf (scale bar: 10 mm). B) Structural model of the micro‐robot, including a front leg, a hind leg with bionic feet, and a miniature electromagnetic linear actuator. C) Performance parameter diagram of the micro linear motor. D) Analysis of the robot's locomotion mode with bionic feet under 5 and 100 Hz driving frequencies.

**Figure** **1**A presents a size comparison between MRBF and a natural leaf, highlighting its miniature scale. As shown in Figure 1B, MRBF has a body length of 7.5 mm and a mass of only 200 mg. Its structure comprises a front leg, a hind leg, a limiting structure, and a micro electromagnetic linear motor. The electromagnetic linear motor serves as the actuation source for the micro‐robot and consists of a precisely wound inductor, a pair of permanent magnets, and a magnetic core. The actuator enables relative motion between the inductor and magnetic core under a low current, achieving a displacement stroke of up to 0.5 mm. When an effective current is applied, the mover transfers the generated relative displacement to the robot's front and hind legs.

The magnetic core adopts an E‐shaped structural design, which helps to reduce magnetic leakage in the actuation unit.^[^ ^38^ ^]^ To convert electrical energy into mechanical energy, the electromagnetic linear motor relies on a precisely wound coil. Under comparable thrust and stroke conditions, the actuator measures only 3 mm × 2 mm × 1.5 mm, significantly smaller than those in comparable micro‐robotic systems.^[^ ^22^ ^]^ This demonstrates the superior performance of the actuation system. As shown in Figure 1C, the electromagnetic linear motor exhibits excellent linearity, generating a thrust of 18.9 mN under a 1 A current input.

Regarding its locomotion mechanism, the micro‐robot generates forward motion by utilizing the inclination of the electromagnetic linear motor, which drives the coordinated movement of the front and hind legs. The reciprocating motion produces a difference in frictional forces between the forward and backward strokes, thereby propelling the robot forward. We conducted an analysis of the robot's actual locomotion mode. High‐speed camera recordings reveal that the micro‐robot exhibits distinct movement patterns at low and high frequencies, with a transition threshold ≈60 Hz. At low driving frequencies, the motion resembles inchworm‐like crawling, while at high frequencies, the behavior shifts to a kangaroo‐like continuous jumping motion (Figure 1D; Movie S1, Supporting Information).

At low frequencies (e.g., 5 Hz), the micro‐robot exhibits a stable and periodic locomotion pattern, consistent with our previous findings.^[^ ^23^ ^]^ The hind legs remain stationary owing to relatively high static friction. The linear motor lifts the front legs upward at an angle, mimicking inchworm‐like movement. The front leg lands smoothly, and the hind leg resets with ease. Gravity assists in maintaining the stability of the motion cycle and enables controllable displacement. In this quasi‐static regime, each locomotion cycle includes well‐defined stance and swing phases. During the stance phase, the hind leg maintains firm ground contact, anchoring the body while the front leg swings forward. Once the front leg lands, the hind leg enters its swing phase to reset. The relatively long contact time and minimal inertial influence ensure precise and repeatable steps. Moreover, the symmetrical alternation of frictional contact between the front and hind legs maximizes the utilization of static friction, resulting in high stability and accurate displacement control. This low‐frequency gait is particularly advantageous for applications requiring controlled navigation over uneven or delicate surfaces.

At high frequencies (e.g., 100 Hz), the robot's locomotion diverges from the low‐frequency periodic behavior and instead adopts a jumping mode, similar to that observed in SMALLbug at 20 Hz.^[^ ^22^ ^]^ In this high‐frequency regime, the front leg repeatedly lifts off the ground owing to strong motor‐induced impulses. Notably, at the end of each cycle, the front leg may remain airborne, and as the next cycle begins, it is again propelled forward without making ground contact. This intermittent contact results in very brief interaction times and relatively low contact forces, as shown in the yellow‐highlighted region of Figure 1D. Such a pattern substantially reduces the frictional drag typically associated with the front leg, minimizing energy loss during ground interaction. Throughout the reciprocating motion, the front leg remains predominantly elevated, contributing minimally to frictional resistance. In contrast, the hind leg maintains more frequent and forceful contact with the ground, generating counterforces through high‐frequency friction. This dynamic enables the hind leg to act as the primary driver, efficiently converting rapid motor impulses into forward propulsion.

### 2.2 Bionic Feet Incorporation

The ability of tree frogs to adhere to wet and smooth surfaces is largely attributed to the unique microstructures on their toe pads.^[^ ^39^ ^]^ These toe pads are primarily composed of densely packed arrays formed by hexagonal prisms and grooves, which allow liquids to rapidly spread and fill the grooves across the surface.^[^ ^40^ ^]^ When subjected to pressure, these grooves facilitate the quick transfer of water, enabling the structure to maintain a critical level of friction under varying pressure and surface conditions. This results in enhanced friction and adaptability in complex environments.^[^ ^41^ ^]^

**Figure 2:**
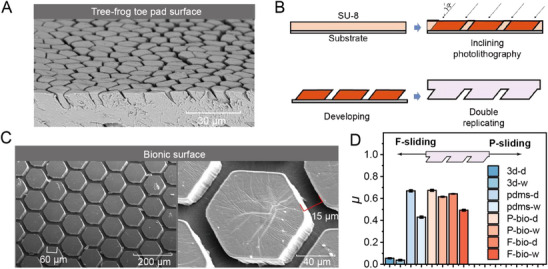
Bionic foot processing flow and characterization. A) Scanning electron microscopy (SEM) images of the natural bionic foot structures in tree frogs. B) Fabrication process of the artificial bionic foot. C) SEM images of the fabricated bionic feet, showing hexagonal structures with a side length of 60 µm, a height of 30 µm, groove dimensions of 15 µm, and an inclination angle of 20°. D) Coefficient of friction under various surfaces and conditions.

To improve the terrain adaptability, we aim to enhance its locomotion performance by incorporating a tree frog‐inspired microstructure into its toe pads. The bionic feet designed in this study closely replicate the microstructure of tree frog toe pads, which are known for their exceptional adhesion to wet and inclined surfaces. The SEM image of a tree frog's toe pad (**Figure** **2**A) reveals densely packed hexagonal prisms and grooves, which enable rapid water drainage and generate high friction under varying pressure conditions. Inspired by this biological architecture, we designed and fabricated high‐friction bionic feet with anisotropic frictional properties.

In early studies, the frictional anisotropy and adaptability of various polygonal pillar patterns—including quadrangular, triangular, rhomboid, circular, and multiple hexagonal configurations—were systematically investigated under wet conditions.^[^ ^42^, ^43^ ^]^ While these designs can introduce some degree of adhesion enhancement, the anisotropic friction effect is not universal across all geometries. Specifically, circular or square arrays typically generate isotropic or weakly anisotropic friction because their symmetry lacks a preferred direction for differential resistance. In contrast, hexagonal arrays, especially when combined with an inclination angle, exhibit distinct directional frictional properties owing to their asymmetric contact line distribution and efficient liquid drainage pathways.

Experimental comparisons confirmed that the inclined hexagonal prism pattern provided the highest friction coefficient and the most pronounced anisotropy on wet surfaces, outperforming quadrangular and circular counterparts. This performance advantage is attributed to two synergistic mechanisms: i) Enhanced drainage of the interfacial liquid through interconnected groove channels, which suppresses hydrodynamic lubrication, and ii) Stronger capillary forces of multiple liquid films due to the pillar inclination. Thus, while anisotropy is not an inherent property of all micro‐patterns, the hexagonal inclined structure emerges as the optimal geometry to achieve strong and stable anisotropic friction, which directly benefits the locomotion efficiency and adaptability of our microrobot.

As shown in Figure 2B, the bionic feet were fabricated using microelectromechanical systems (MEMS) technology. The direction with the same tilt angle is defined as the proximal direction, abbreviated as the *P* direction, and the opposite is referred to as the front direction, abbreviated as the *F* direction. As shown in Figure 2C, the hexagonal structure has a side length of 60 µm, a height of 30 µm, groove dimensions of 15 µm, and an inclination angle of 20°.

Through experiments, as shown in Figure 2D, we find that the 3D‐printed parts exhibit a coefficient of friction (*µ*) of 0.053 on dry surfaces and 0.038 on wet surfaces. In contrast, the smooth polydimethylsiloxane (PDMS) surface shows a *µ* of 0.67 on dry surfaces and 0.43 on wet surfaces. The bionic PDMS foot exhibits a *µ* of 0.674 in the *P* direction and 0.642 in the *F* direction on dry surfaces, and a *µ* of 0.615 in the *P* direction and 0.493 in the *F* direction on wet surfaces. Owing to the inclined characteristics of the prismatic array, the bionic foot demonstrates anisotropic frictional behavior on wet surfaces, which is conducive to effective movement.

These results indicate that the bionic feet offer a marginal improvement over the smooth PDMS surface under dry conditions and a significant enhancement compared to 3D‐printed parts. More importantly, under wet conditions, the bionic feet show substantial improvement in friction performance relative to the smooth PDMS surface. Additionally, owing to the inclined prismatic structure, the bionic foot exhibits pronounced anisotropy between the *P* and *F* directions, which benefits the robot's locomotion.

Based on the tree frog toe pad microstructure, the bionic high‐friction feet possess unique advantages, including reduced sensitivity to wet environments and pronounced directional frictional anisotropy. These features provide a solid foundation for applying bionic feet in complex terrains, enabling excellent performance across various operational scenarios.

### 2.3 Bionic Feet Enhancement

**Figure 3:**
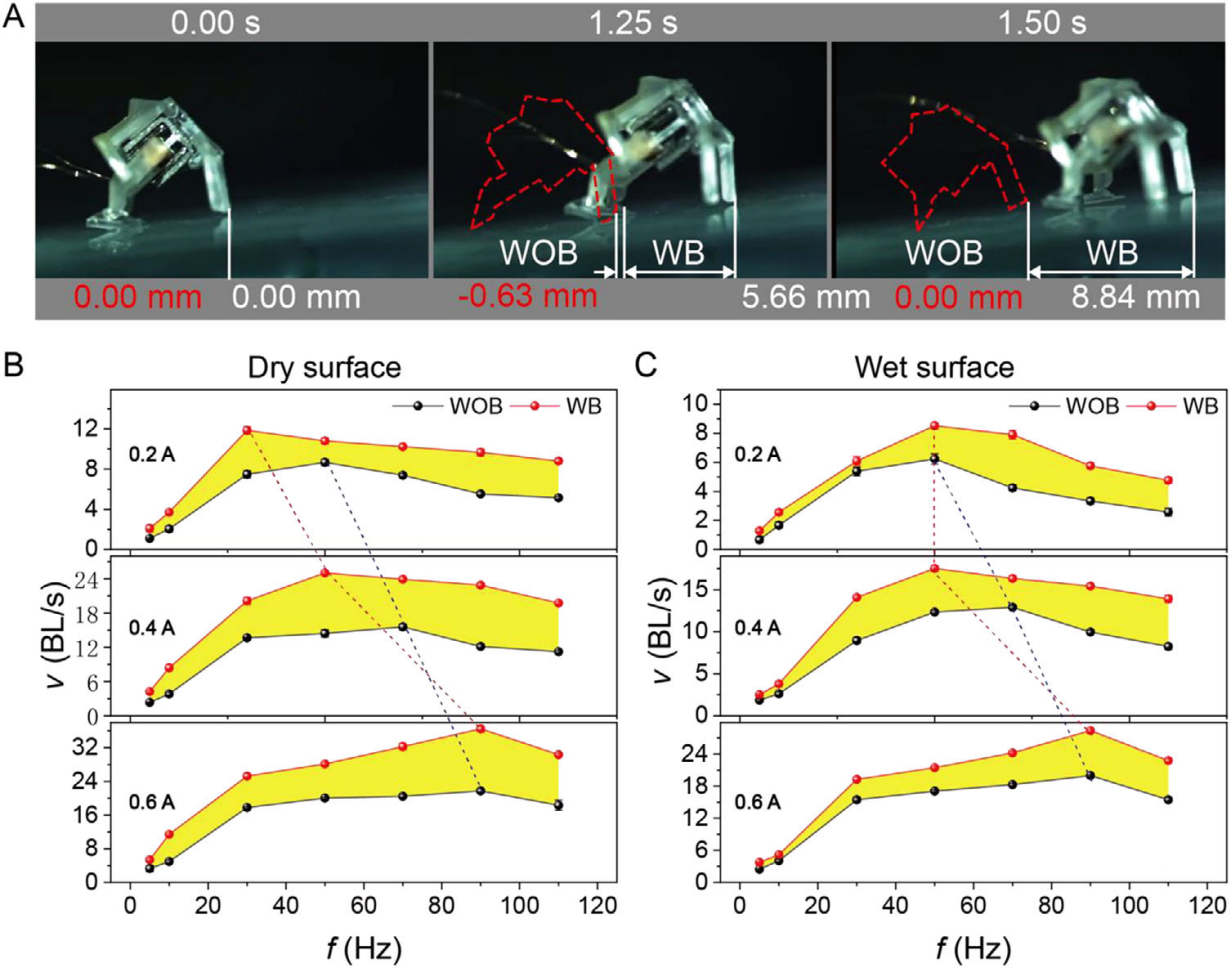
Performance improvements resulting from the integration of bionic feet. A) Performance comparison of micro‐robots with and without bionic feet. B) Variation in robot speed under different currents and frequencies on dry surfaces. C) Variation in robot speed under different currents and frequencies on wet surfaces.

Comparative experiments were conducted to evaluate the impact of bionic feet on the robot's locomotion performance (**Figure** **3**A; Movie S2, Supporting Information). A smooth glass substrate was selected as the testing platform to highlight performance differences. Without bionic feet, the robot failed to achieve forward motion within 1.5 s and exhibited minor backward slippage. In contrast, after being equipped with bionic feet, the robot successfully moved forward by 8.84 mm over the same duration, demonstrating a significant improvement in locomotion capability.

Figure 3B,C presents the experimental results for the robot's moving speed under various conditions, with the yellow‐highlighted areas indicating the speed enhancement achieved when the robot is equipped with the bionic surface. Each condition was tested three times, and the average displacement was calculated, with corresponding relative errors indicated. Without bionic feet (WOB), the robot exhibited lower moving speeds. In contrast, with bionic feet (WB), the robot achieved significantly greater displacement, demonstrating that the bionic structure substantially enhances dynamic performance.

This improvement in locomotion can be attributed to the significant increase in frictional force provided by the bionic surface. The tree frog‐inspired microstructure introduces anisotropic friction, generating directional resistance that favors forward movement while reducing backward slippage during the retraction phase. The inclined hexagonal prism arrays on the bionic feet increase the real contact area and enable efficient water drainage on wet surfaces, maintaining high adhesion even under reduced normal forces. As a result, the bionic structure enables more effective traction during each actuator stroke, allowing a greater proportion of the motor's input energy to be converted into translational motion. Furthermore, the increased friction stabilizes foot placement during the actuation cycle, minimizing energy loss due to slipping and enabling the robot to operate more efficiently, particularly at higher frequencies. This combination of enhanced grip, directional control, and efficient energy transfer collectively contributes to the observed improvements in speed and locomotion efficiency.

Furthermore, the effects of different substrate types, driving currents, and actuation frequencies are discussed in detail. The experimental results show that the incorporation of bionic feet significantly enhances the robot's motion performance across a range of driving conditions and environments. In dry environments and across all tested current levels (0.2–0.6 A), the robot equipped with bionic feet consistently outperformed the version without them. At a driving current of 0.2 A, the robot reached a peak speed of 12.1 BL s^−1^ at ≈ 30 Hz, with the bionic feet providing a speed enhancement of up to 70% compared to the robot without bionic feet. When the current was increased to 0.4 A, the performance gain from the bionic feet was further amplified, resulting in a 78.5% speed increase at 50 Hz. At 0.6 A, the bionic feet still provided a substantial speed gain of 66.7% at 90 Hz.

Meanwhile, the moving speed of MRBF increased with rising current. Each current level exhibited an optimal operating frequency, indicating that enhanced motor acceleration allows the robot to maintain full‐stroke motion at higher frequencies. As the current increased from 0.2 to 0.6 A, the optimal frequency shifted upward, from 50 to 90 Hz. Notably, under the same current, the optimal frequency of the robot equipped with bionic feet shifted marginally lower owing to the hind legs needing to overcome greater static friction. This frequency stabilized ≈90 Hz.

Tests conducted in wet environments further demonstrated the adaptability of the bionic feet to complex interfaces. Although the presence of water led to an overall speed reduction of 5–15% compared to dry conditions, the bionic feet maintained a significant performance advantage. This was achieved by facilitating rapid water drainage through surface grooves, thereby preserving essential friction. At a driving current of 0.2 A, the bionic feet enabled an 87% increase in speed at 70 Hz compared to the robot without bionic feet. When the current increased to 0.4 A, the bionic feet produced a 42% speed gain at 50 Hz, indicating that their traction optimization mechanism is particularly effective under moderately wet conditions.

At higher currents, water significantly exacerbated the speed attenuation of the robot without bionic feet, resulting in a 20% reduction in speed compared to dry conditions. In contrast, the robot with bionic feet limited the speed attenuation to within 8%, highlighting its superior ability to maintain locomotion efficiency in wet environments. Correspondingly, as the driving current increased, the optimal operating frequency of the system progressively shifted from 50 to 70 Hz and then to 90 Hz.

In summary, in dry environments, high‐frequency driving force is primarily transmitted through enhanced friction. In wet environments, the micro‐groove structures on the surface conduct water away and disrupt the continuous water film to restore effective contact. Overall, in both dry and wet conditions, the bionic feet significantly increase the speed of MRBF, achieving improvements of 40–87% within the current range of 0.2–0.6 A. Moreover, regardless of the presence of bionic feet, the robot's maximum speed consistently increased with rising current.

### 2.4 Performance Characterization

**Figure 4:**
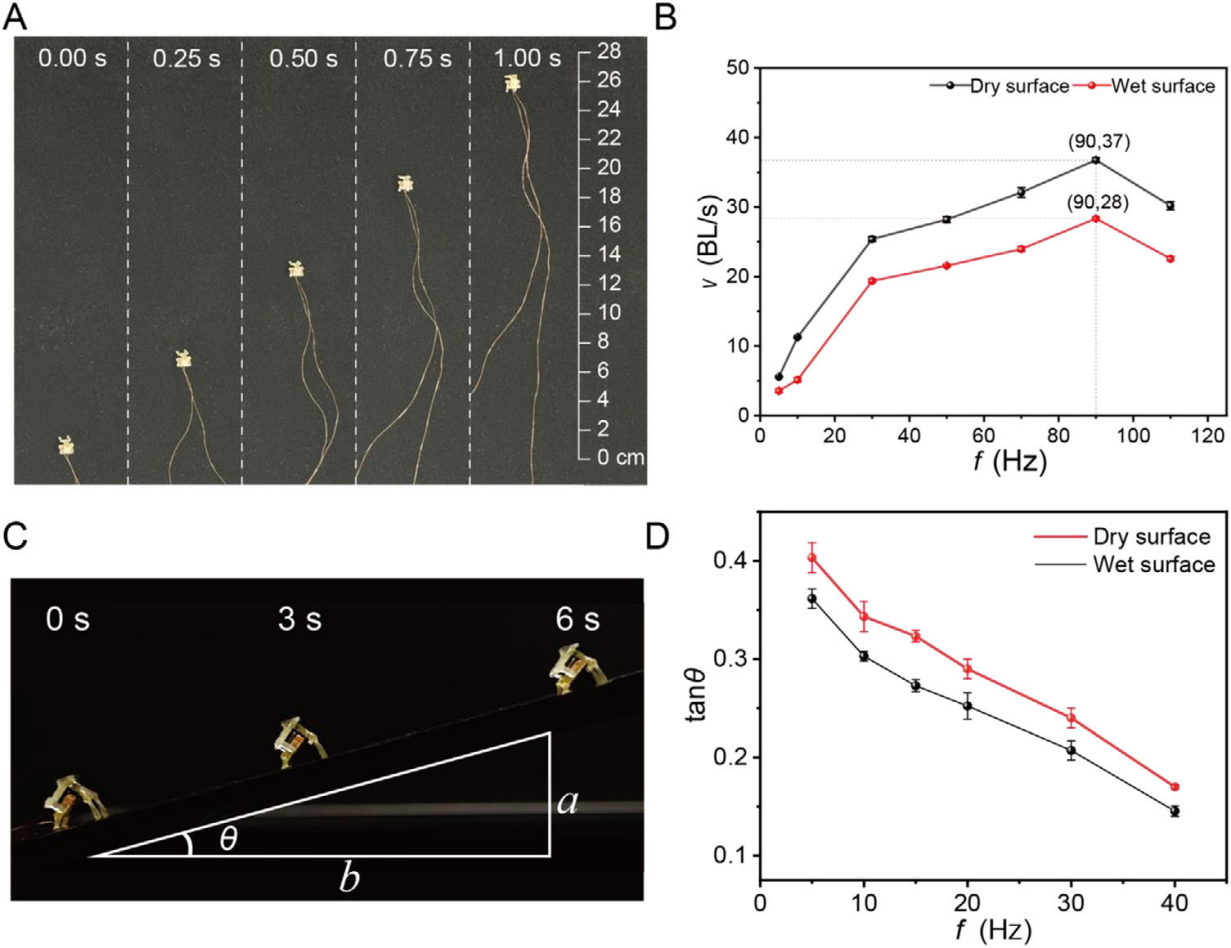
Ultimate performance of single‐unit micro‐robots. A) Optical images of MRBF moving in a straight line. B) Ultimate moving speeds of micro‐robots on dry and wet surfaces. C) Optical images of MRBF climbing a 15° slope. D) Maximum climbing angle of the micro‐robot at different driving frequencies on various surfaces.

This section focuses on the ultimate performance of the MRBF. Moving speed is a critical performance indicator for micro‐robots, reflecting both their agility and adaptability. To evaluate extreme motion capabilities, a series of tests was conducted. As noted in previous sections, the moving speed of the micro‐robot consistently increases with rising driving current. In this study, ultimate speed tests were performed under a fixed maximum current of 0.6 A. Under dry conditions, the MRBF achieved a maximum speed of 39 BL s^−1^ (**Figure** **4**A), while under wet conditions, it maintained a high speed of 28.5 BL s^−1^ (Movie S3, Supporting Information). The optimal frequency for achieving maximum speed was ≈90 Hz (Figure 4B), representing a performance improvement of 20.2 BL s^−1^ compared to an electromagnetically driven micro‐robot of similar size operating under dry conditions.^[^ ^24^ ^]^ Furthermore, the results reveal that as the driving frequency increases, the robot's speed initially rises and then declines. This speed–frequency behavior can be explained as follows: At frequencies up to ≈20 Hz, the motor achieves its full designed stroke during each actuation cycle. In this range, speed increases proportionally with frequency because the stroke remains consistent, and a greater number of full‐displacement cycles occurs per unit time. Between 5 and 30 Hz, the motor's stroke gradually decreases owing to limitations in current supply (e.g., 0.25 A). However, because the number of cycles per second increases, the robot is able to complete more motion cycles within a given time, compensating for the reduced stroke per cycle. As a result, the overall speed continues to increase, although with diminishing returns. Once the frequency exceeds the optimal threshold, stroke reduction becomes significant. Although the actuation frequency continues to rise, the stroke length becomes too small to generate meaningful displacement per cycle. At this point, the diminished stroke outweighs the benefit of increased cycle rate, and the overall speed begins to decrease. Thus, the robot's speed follows a nonlinear response with frequency, peaking at an optimal value where stroke and cycle rate are best balanced. Beyond this point, insufficient stroke limits effective propulsion, resulting in reduced locomotion efficiency.

Moreover, the robot's ability to ascend slopes significantly enhances its motion versatility. To evaluate this, slope‐climbing tests were conducted at various inclinations, as shown in Figure 4C. The vertical axis indicates the MRBF's climbing capability. The current was set at 0.6 A, and the driving frequency was varied to assess performance. The slope surface was constructed from polyvinyl chloride (PVC), with a coefficient of friction (*µ*) of 0.074, simulating a smooth environment. Under dry conditions, the MRBF successfully climbed a 21.8° slope at a frequency of 5 Hz (Figure 4D). On a wet surface, it managed to ascend a 19.5° slope. Furthermore, when tested on a slope with a higher *µ* of 0.55, the robot effectively climbed a 30° incline (Movie S4, Supporting Information). This climbing performance surpasses that of a previously reported robot with a 5 mm body length by 10°, and another micro‐robot with a 20 mm body length by 25°.

**Figure 5:**
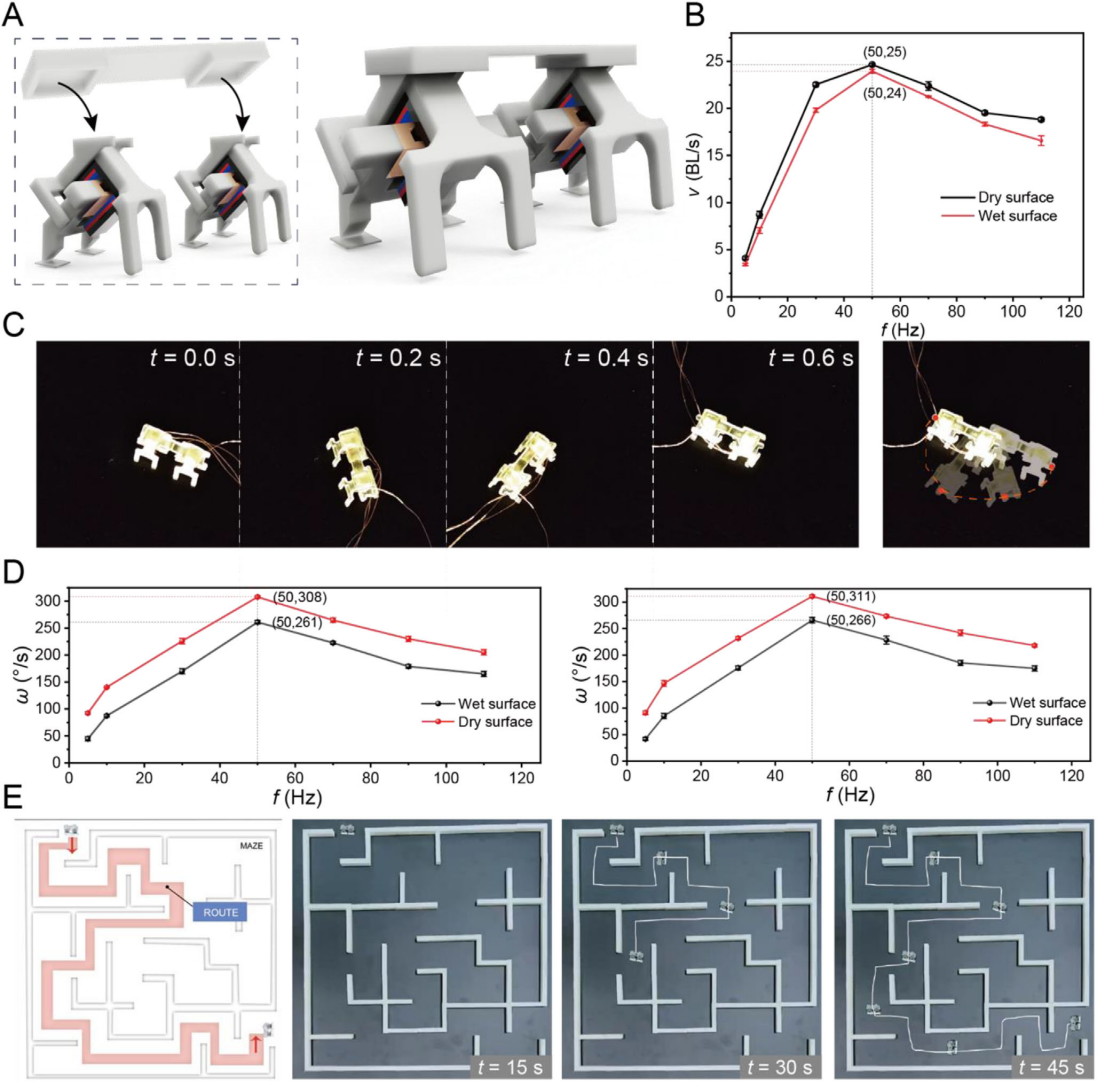
Ultimate performance of dual‐MRBF. A) Configuration of the dual‐MRBF system. B) Maximum moving speed of dual micro‐robots on dry and wet surfaces. C) Photographs of the dual micro‐robot executing turns. D) Left and right turning angular velocities of dual‐MRBF across driving frequencies on different surfaces. E) Dual‐MRBF navigating through a maze, taking 45 s to complete the task.

Turning ability is another critical factor in evaluating the agility of micro‐robots. As shown in **Figure** **5**A, two MRBF units were connected via a linking component and positioned side by side. By independently controlling the left and right actuators, open‐loop experiments were conducted to assess turning performance. The results demonstrate that, when asymmetric inputs were applied to the left and right actuators, the robot reliably changed its direction of movement, confirming its agility under differential drive.

The moving speed of the dual‐MRBF was first evaluated. Similar to the single‐robot results, the dual‐MRBF exhibited a trend in which speed increased with frequency, peaked at an optimal point, and then declined (Figure 5B). Additionally, at a fixed frequency, speed consistently increased with rising current. Figure 5B shows data collected at a driving current of 0.6 A, highlighting the maximum speed of the dual‐MRBF across various frequencies. The optimal speed was consistently observed at 50 Hz for both dry and wet surfaces. The difference in optimal frequency between the single MRBF (90 Hz) and the dual‐MRBF system (50 Hz) is primarily attributed to the interaction effects between the two robots in the coupled configuration. In the dual setup, the two robots are mechanically linked and act as a single unit, introducing additional mechanical loads that influence the overall motion dynamics. These interaction effects, including inter‐robot friction and increased mechanical resistance, impose constraints that reduce system agility, resulting in a lower optimal operating frequency compared to the single‐robot case. The coupling introduces alignment challenges and additional energy losses that necessitate a reduced frequency to maintain coordinated and efficient movement. In contrast, the single MRBF operates independently, with fewer mechanical constraints and greater freedom of motion, which allows it to achieve a higher optimal frequency of 90 Hz. Although the robot achieved marginally higher speeds on dry surfaces than on wet ones, the performance enhancement provided by the bionic feet remained evident under both conditions. Compared to the single‐robot configuration, the dual‐MRBF exhibited a marginally reduced maximum speed, which is attributed to minor discrepancies between the two motors. These discrepancies can lead to minor misalignments in movement, reducing efficiency and overall speed. Additionally, the mechanical coupling between the two units introduces extra resistance and coordination demands, further contributing to the marginal reduction in speed relative to the single‐robot system.

To assess turning performance, left and right turning speeds were measured on standard PVC pads (Figure 5C; Movie S5, Supporting Information). At a fixed current, the angular turning velocity increased with rising input frequency on one side, peaking and then gradually decreasing (Figure 5D). The dual‐MRBF exhibited balanced performance for both left and right turns, with optimal results achieved at 50 Hz. Specifically, left turns reached a peak angular velocity of 311° s^−1^ on dry surfaces and 266° s^−1^ on wet surfaces. For right turns, the robot achieved 308° s^−1^ on dry surfaces and 261° s^−1^ on wet surfaces. This dual‐MRBF system not only exceeds the 180° s^−1^ angular velocity of similarly sized soft robots ^[^ ^44^ ^]^ but also addresses the directional imbalance seen in other designs, such as the SMARTI robot, which exhibited an 84° s^−1^ discrepancy between left and right turns.^[^ ^45^ ^]^ Moreover, the dual‐MRBF maintains consistent left–right turning behavior and good stability across environments. Its turning radius is only half the body width (7 mm), with rotation occurring around the leg, and remains consistent during right turns.

To further demonstrate the capability of the proposed system in challenging confined environments, a maze‐like path was constructed to emulate the complex and tortuous internal geometries typically encountered in aero‐engine cavities (Figure 5E; Movie S6, Supporting Information). The integration of a dual‐motor configuration endows the MRBF with precise steering control, enabling it to change direction smoothly without disrupting its overall motion state. With a turning radius comparable to its own body width and an angular velocity exceeding 300° s^−1^, the robot achieves excellent maneuverability, which is essential for navigation within narrow and curved passages.

Under open‐loop control, the dual‐MRBF successfully navigated through the maze and reached the exit within 45 s, demonstrating reliable path‐following and high positional accuracy. This experiment validates the robot's strong environmental adaptability and motion stability in highly constrained spaces. The results also highlight that, unlike conventional endoscopes which are limited to passive insertion along pre‐defined channels, the MRBF can actively explore, turn, and reorient itself within complex 3D environments. Such mobility further substantiates its potential for autonomous inspection in compact aero‐engine architectures where flexible endoscopic tools face intrinsic geometric limitations.

### 2.5 Performance Comparison

**Figure 6:**
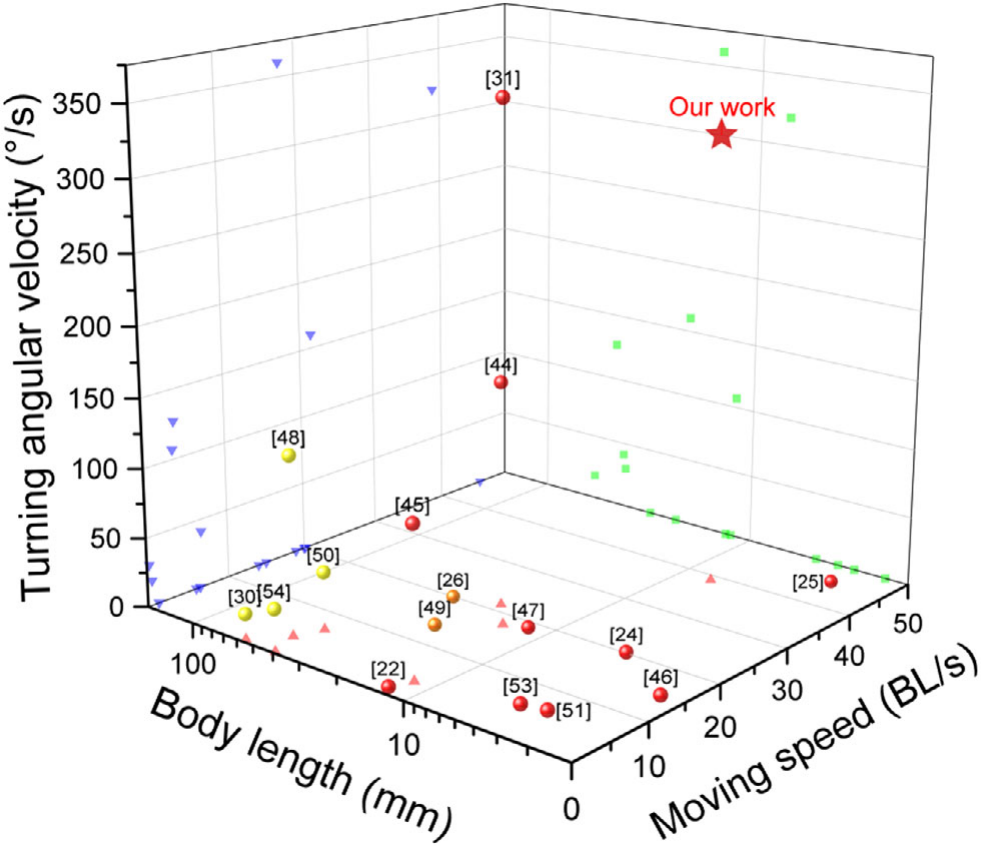
Comparison of body length, moving speed, and turning angular velocity among previously reported robots. Data are available in Table 1.

The MRBF we designed and fabricated exhibits superior overall performance at the same size level, surpassing similar micro‐robots in several key performance metrics. As shown in **Figure** **6**, we conduct a comparative analysis of body length, moving speed, and turning angular velocity across various micro‐robots, highlighting the advantages of our design in these critical areas. MRBF demonstrates an optimized balance among three core performance metrics, size, speed, and controllability, offering exceptional integrated capabilities. This makes it particularly well‐suited for complex tasks in confined or constrained environments. While other micro‐robots may excel in individual metrics (e.g., 2.5 mm in length,^[^ ^46^ ^]^ maximum speed of 46 BL s^−1^,^[^ ^21^ ^]^ or turning velocity over 360° s^−1^.^[^ ^31^ ^]^), our design stands out for its high level of integration at the microscale. It effectively combines compact size, high maneuverability, and precisely controllable steering. Additionally, it can crawl stably on a 30° incline, further enhancing its adaptability and expanding its potential for deployment in complex environments.

Cost of transport (CoT) is used to measure the efficiency of robot crawling, and it is calculated as follows:

(1) $$\mathrm{CoT}=\frac{P}{\mathrm{mgV}}$$

The CoT for the robot proposed in this study ranges from 226.5 to 627 under different inputs. For instance, when the robot achieves its maximum crawling speed (400 mA, 50 Hz), with a power consumption of 0.16 W, the CoT is 418.7. Because of the inevitable heating of the coils, CoT is not an advantage for robots based on electromagnetic principles. However, compared with the CoT of some previously reported robots of different designs, this value is acceptable (Ref.,^[^ ^44^ ^]^ CoT of < 1000; Ref.,^[^ ^54^ ^]^ CoT of 1670).

**Table** **1** compares the performance metrics of micro‐robots with various actuation methods reported in recent studies. MRBF exhibits superior controllable high speed and terrain adaptability at the microscale. Moreover, the robot operates at a current of 0.6 A (with a theoretical voltage requirement of 1 V), highlighting its potential for future wireless operation.

**Table 1:** Performance comparison of robots with different actuation methods in recent years.

References	Actuator	Body length [mm]	Terrain adaptability	Moving speed [BL s^−1^]	Turning angular velocity [° s^−1^]	Gradeability [°]
[44]	Electromagnetic	20	Dry/wet	21.5	160	25
[24]	Electromagnetic	5	Dry	20.2	–	10
[47]	Electromagnetic	12.3	Dry	18.9	–	3.2
[31]	Electromagnetic	15	Dry	18	360	6
[29]	Electromagnetic	9/20	Dry/wet	35	160	–
[45]	SMA	12	Dry	3.54	107	–
[22]	SMA	13	Dry	1.3	–	–
[48]	Piezoelectric	45	Dry	3.8	127	–
[49]	Piezoelectric	22.5	Dry	13.91	–	–
[26]	Piezoelectric	30	Dry	20	–	15.6
[50]	Piezoelectric	41	Dry	6.7	41	–
[46]	External Magnetic	2.5	Dry	14.9	–	–
[51]	External Magnetic	4	Dry	6.4	–	–
[52]	External Magnetic	17	Dry/wet	0.03	–	> 50
[25]	Electrostatic	3.4	Dry	46	–	–
[53]	Electrostatic	5	Dry	5.9	–	–
[54]	DEA	40	Dry	0.3	30	–
This work	Electromagnetic	7.5	Dry/wet	39	260	30

Micro‐robots have demonstrated substantial potential for the inspection of complex equipment, particularly in scenarios where conventional methods are limited or inefficient. With its compact size, high maneuverability, and strong adaptability, the MRBF can navigate through narrow gaps and harsh conditions within intricate instruments to conduct high‐precision detection tasks.

**Figure 7:**
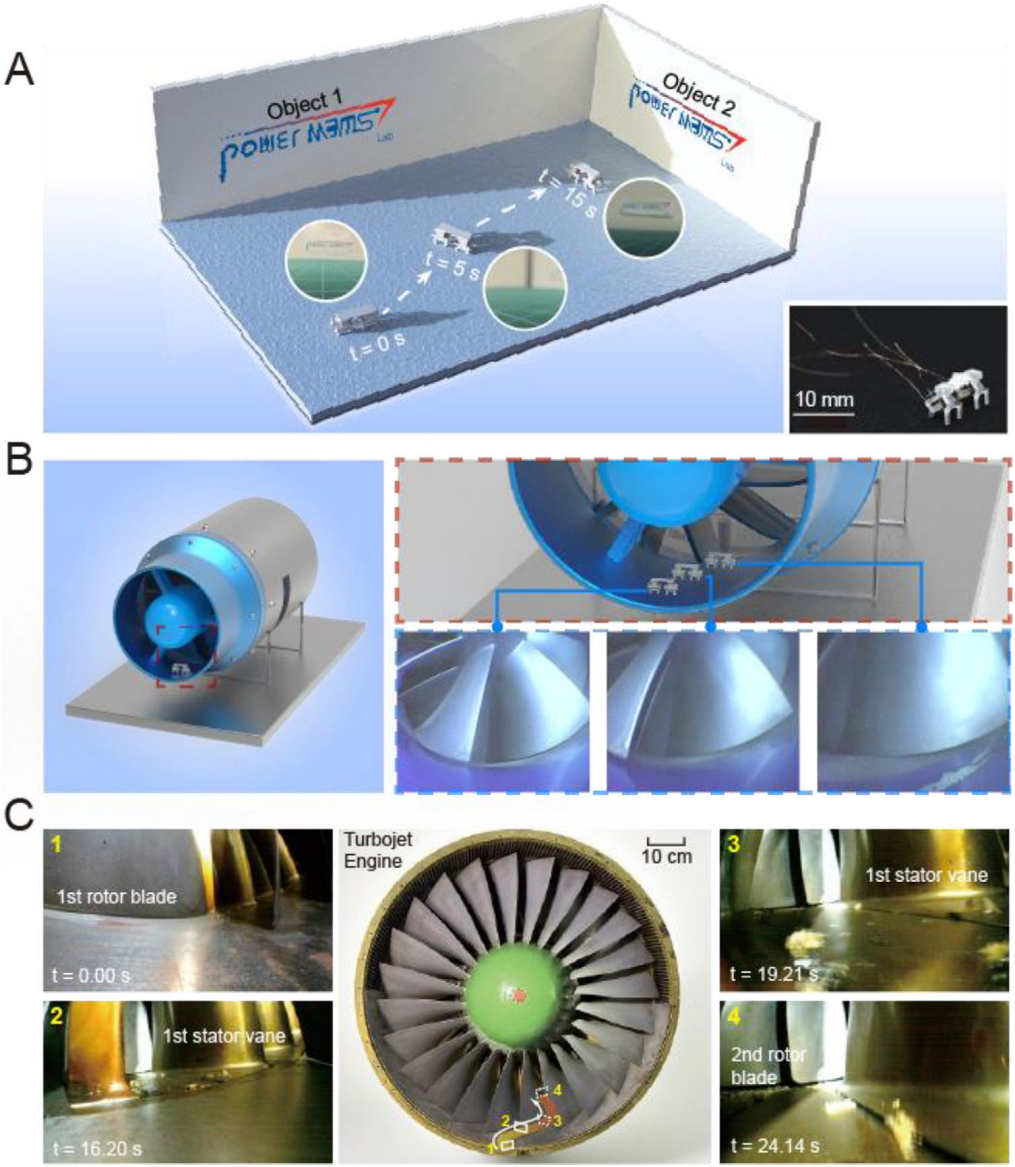
Demonstration of application scenarios for the micro‐robot equipped with a micro‐camera. A) Controlled turning of micro‐robots with cameras on laboratory desks. Real‐time images transmitted by the camera are shown in the inset. B) Dual‐MRBF equipped with cameras navigate into micro‐aero‐engines and transmit real‐time visual data for inspection. C) Dual‐MRBF enters a large‐scale turbojet and captures real‐time images of 1st rotor blade, 1st stator vane and 2nd rotor blade.

To verify this potential, the robot was equipped with an OV6916 micro‐camera and tested under laboratory conditions for autonomous steering and real‐time image transmission (**Figure** **7**A; Movie S7, Supporting Information). Experimental results show that the robot can move flexibly and collect clear image data, enabling effective visual inspection inside complex equipment.

The application of micro‐robots in the internal inspection of aero‐engines was also examined. As shown in Figure 7B, the robot was guided into the compressor section of an FW‐80 series micro turbojet engine via the air intake (Movie S8, Supporting Information). The test platform is a compact, small‐scale engine with a total length of 48 cm, an intake diameter of 13 cm, and a maximum body diameter of 20 cm. These tight spatial constraints pose significant challenges for conventional inspection tools, rendering them unsuitable for internal navigation. During the crawling process, the MRBF moved stably along the narrow intake path and successfully reached the target detection region. Upon arrival, the onboard micro‐camera captured real‐time images and enabled visual recognition of internal structural features. To further demonstrate the applicability of MRBF in aero‐engine inspection, an additional experiment was conducted using a large‐scale turbojet engine. In this extended scenario, the MRBF is able to navigate deeper into the compressor section for internal inspection. As shown in Figure 7C, MRBF successfully bypasses multiple rotor and stator blade rows—starting from the first‐stage rotor blades (t = 0.00 s), moving past the first‐stage stator vanes (t = 16.20 and 19.21 s), and finally reaching the second‐stage rotor blades (t = 24.14 s). This sequence illustrates the ability of MRBF to maneuver through narrow inter‐blade passages and perform close‐range inspections of internal engine components. Its compact design, strong electromagnetic actuation, and friction‐enhancing bionic feet allowed it to traverse narrow, curved surfaces with high stability. This experiment directly addresses the challenge of inspecting internal blade structures, which is significantly more complex than examining primary blades. The MRBF successfully performed real‐time imaging within a confined engine environment, reinforcing its utility for future maintenance, structural health monitoring, and inspection of aerospace systems with limited accessibility.

## 3 Conclusion

This study presents a controllable 7.5 mm micro‐robot with bionic feet, driven by an electromagnetic linear motor, referred to as MRBF. Utilizing advanced fabrication techniques, the motor delivers considerable thrust despite its compact dimensions, enabling efficient locomotion at the microscale. The MRBF's locomotion mechanism operates through modulation of the inclination angle of motor, which induces differential frictional forces between the front and hind legs, driving forward motion.

A central innovation of this work lies in the integration of bionic feet inspired by the adhesive toe pads of tree frogs. These structures significantly enhance the grip of the hind legs, resulting in improved terrain adaptability and increased locomotion speed. Experimental characterization reveals a substantial increase in the coefficient of friction, with pronounced anisotropic behavior between the front and rear contact interfaces. As a result, the robot equipped with bionic feet achieves a maximum speed of 39 BL s^−1^ on dry surfaces, representing a 75% improvement over the baseline configuration without the bionic enhancement. On wet surfaces, MRBF maintains a speed of 28.5 BL s^−1^, offering a 40% improvement compared to the non‐bionic version.

In terms of climbing performance, the addition of bionic feet improves slope‐climbing capability by 98%, allowing the robot to ascend inclines of up to 30°. This advancement indicates the robot's enhanced adaptability to complex and uneven terrains, an essential attribute for real‐world deployment. To further improve maneuverability, the study introduces a modular dual‐MRBF assembly, enabling responsive and controllable turning. The system achieves angular velocities of 311 and 308° s^−1^ for left and right turns, respectively, under dry conditions. Even on wet surfaces, the dual configuration maintains high turning performance, reaching 266 and 261° s^−1^ for left and right turns, respectively.

Collectively, these results demonstrate the MRBF's considerable progress in speed, terrain adaptability, and turning agility, highlighting its suitability for operation in complex or constrained environments. The practical utility of MRBF is further validated through successful deployment in the intake duct of a micro turbojet engine, where it performs real‐time imaging and internal inspection. This application indicates MRBF's potential in industrial endoscopy, fault detection in confined spaces, and disaster‐response scenarios where conventional inspection tools are ineffective or infeasible.

Although the MRBF demonstrates remarkable speed, high maneuverability, and strong terrain adaptability, several limitations persist. Specifically, its prolonged operation under high current levels (exceeding 0.6 A) may induce Joule heating within the electromagnetic actuator, leading to temperature rise, reduced efficiency, and potential long‐term performance drift. Future studies will be focused on improving the thermal management of the actuator, such as through optimized coil materials, magnetic circuit redesign, and intermittent drive strategies, to further enhance the device performance. Moreover, for stable operation, the present system relies on a tethered power supply, which inherently constrains its range of motion and limits its deployment in narrow or enclosed spaces. Therefore, future investigations will be concentrated on integrating lightweight, high‐energy‐density micro‐batteries and on exploring wireless power transfer methods, such as magnetic resonance coupling, with the aim of achieving a fully untethered configuration and thus realizing autonomous and continuous operation.

The system proposed in the present study advances the field of micro‐robotics, offering a versatile platform for precision inspection, maintenance, and rescue operations in challenging and space‐limited environments. In the future, we will aim toward further optimizing and overcoming these limitations to realize a more robust, self‐powered, and application‐ready micro‐robotic system.

## 4 Experimental Section

### Structural Design and Fabrication of the Micro‐Robot

The legs of the robot were fabricated using a Creality Halot‐Ray 3D printer, employing ultraviolet‐sensitive resin as the printing material. The micro‐motor consists of an unoriented silicon steel core, two N48M permanent magnets, and a coil. The coil substrate was produced via high‐precision photocurable resin printing, onto which 0.08 mm diameter enameled copper wire was tightly wound under controlled tension. The hind legs were directly affixed to the 3D‐printed coil structure using adhesive glue. The bionic foot was bonded to the hind leg using PDMS glue (J‐807 instant adhesive). After curing, a 3 mm × 3 mm bionic foot was obtained via laser cutting (ZK‐ZW‐5 W).

### Structural Design and Fabrication of the Bionic Feet

Inclined photolithography was used to create an array of inclined prismatic microstructures on a silicon wafer. The wafer surface was subsequently fluorinated with 1H, 1H, 2H, 2H‐perfluorodecyltrimethoxysilane under high‐temperature vacuum conditions to modify surface properties. The bionic frictional surface was then replicated twice using PDMS with a prepolymer‐to‐curing‐agent ratio of 10:1. Finally, the replicated surface underwent oxygen plasma treatment to induce hydrophilicity, resulting in bionic feet with anisotropic high‐friction characteristics.

### Surface Friction Coefficient Measurement

The friction coefficient (*µ*) was measured by pressing a coverslip against the test surface and pulling it at a constant velocity. The peak force during this motion was recorded using a high‐precision dynamometer (SH‐5N, NSCING) and used to calculate the friction coefficient.

### MRBF Performance Testing

To minimize interference from wiring, wires ≈1 m in length and 0.08 mm in diameter were used. The robot was powered by a pulse power supply (MD30‐B). Motion capture was performed using a high‐speed camera (Photron Nova S12). Each velocity measurement was repeated three times, and the average value was reported. Except for the data shown in Figure 3A, all flat‐surface tests were conducted on PVC cutting boards. Supplementary S1

## Conflict of Interest

The authors declare no conflict of interest.

## Supporting information



Supporting Information

Supplementary Movie 1

Supplementary Movie 2

Supplementary Movie 3

Supplementary Movie 4

Supplementary Movie 5

Supplementary Movie 6

Supplementary Movie 7

Supplementary Movie 8

## Data Availability

The data that support the findings of this study are available from the corresponding author upon reasonable request.
