# Supplementary material for: High‐Speed, Maneuverable, and Terrain‐Adaptive Micro‐Robot with Tree Frog‐Inspired Bionic Feet
Source: Adv Sci (Weinh). 2025 Nov 26;13(7):e14807. doi: 10.1002/advs.202514807 (PMC12866859; doi:10.1002/advs.202514807)
Supplement: Supplementary file 1 — Supporting Information [file ADVS-13-e14807-s005.docx]

Supporting Information

High-Speed, Maneuverable, and Terrain-Adaptive Micro-Robot with Tree Frog-Inspired Bionic Feet

Weizhi Zhao, Shijia Li, Kaiwen Zhang, Wanyu Zhang, Danyang Liu, Weidong Fang, Yanxin Zhai, Kaibo Lei, Liwen Zhang, Huawei Chen, and Tiantong Xu*

Movie S1. High-speed camera footage of MRBF driven at 5 and 100 Hz.

Movie S2. Comparison between micro-robots with and without bionic feet.

Movie S3. MRBF locomotion on dry and wet surfaces at high speed.

Movie S4. Slope-climbing performance of MRBF and dual-MRBF.

Movie S5. Left and right turning motions of the dual-MRBF.

Movie S6. Dual-MRBF navigating through a maze in 45 s.

Movie S7. Dual-MRBF equipped with a micro-camera captures a predetermined target under laboratory conditions.

Movie S8. Dual-MRBF equipped with a micro-camera enters the intake passage of the FW-80 engine for real-time inspection and imaging.
